# Localized redox relays as a privileged mode of cytoplasmic hydrogen peroxide signaling

**DOI:** 10.1016/j.redox.2017.01.003

**Published:** 2017-01-06

**Authors:** Rui D.M. Travasso, Fernando Sampaio dos Aidos, Anahita Bayani, Pedro Abranches, Armindo Salvador

**Affiliations:** a Centro de Física da Universidade de Coimbra (CFisUC), Department of Physics, University of Coimbra, Coimbra, Portugal; b Department of Physics & Mathematics, School of Science & Technology, Nottingham Trent University, UK; c Department of Life Sciences, University of Coimbra, Coimbra, Portugal; d Center for Neuroscience and Cell Biology, University of Coimbra, Portugal; e CQC, Department of Chemistry, University of Coimbra, Portugal

**Keywords:** Peroxiredoxins, Redox signaling, Hydrogen peroxide, Redox relays, Mitogenic signaling

## Abstract

Hydrogen peroxide (H_2_O_2_) is a key signaling agent. Its best characterized signaling actions in mammalian cells involve the early oxidation of thiols in cytoplasmic phosphatases, kinases and transcription factors. However, these redox targets are orders of magnitude less H_2_O_2_-reactive and abundant than cytoplasmic peroxiredoxins. How can they be oxidized in a signaling time frame? Here we investigate this question using computational reaction-diffusion models of H_2_O_2_ signaling. The results show that at H_2_O_2_ supply rates commensurate with mitogenic signaling a H_2_O_2_ concentration gradient with a length scale of a few tenths of μm is established. Even near the supply sites H_2_O_2_ concentrations are far too low to oxidize typical targets in an early mitogenic signaling time frame. Furthermore, any inhibition of the peroxiredoxin or increase in H_2_O_2_ supply able to drastically increase the local H_2_O_2_ concentration would collapse the concentration gradient and/or cause an extensive oxidation of the peroxiredoxins I and II, inconsistent with experimental observations. In turn, the local concentrations of peroxiredoxin sulfenate and disulfide forms exceed those of H_2_O_2_ by several orders of magnitude. Redox targets reacting with these forms at rate constants much lower than that for, say, thioredoxin could be oxidized within seconds. Moreover, the spatial distribution of the concentrations of these peroxiredoxin forms allows them to reach targets within 1 μm from the H_2_O_2_ sites while maintaining signaling localized. The recruitment of peroxiredoxins to specific sites such as caveolae can dramatically increase the local concentrations of the sulfenic and disulfide forms, thus further helping these species to outcompete H_2_O_2_ for the oxidation of redox targets. Altogether, these results suggest that H_2_O_2_ signaling is mediated by localized redox relays whereby peroxiredoxins are oxidized to sulfenate and disulfide forms at H_2_O_2_ supply sites and these forms in turn oxidize the redox targets near these sites.

## Highlights

• 2-Cys peroxiredoxins impose strong cytoplasmic and transmembrane H_2_O_2_ gradients.
• Maximal cytoplasmic H_2_O_2_ concentrations are insufficient to oxidize Tyr phosphatases.
• Prx inhibition modestly increases maximal H_2_O_2_ concentration, blunts gradients.
• Sulfenate and disulfide Prx strongly exceed H_2_O_2_ concentrations, are also localized.
• Prx recruitment causes modest local H_2_O_2_ depletion, helps Prx outcompete H_2_O_2_.

## Graphical abstract

**Figure:**
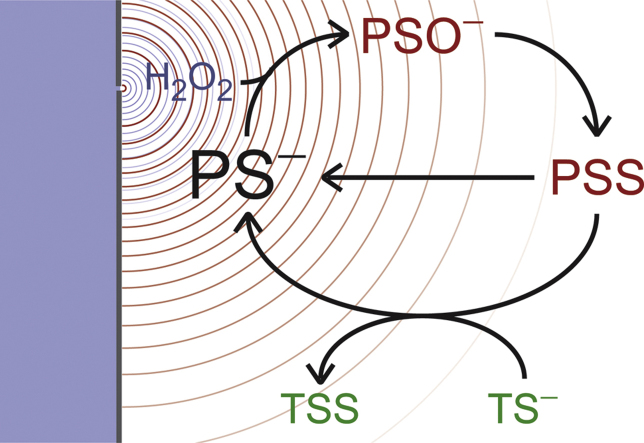

## 1 Introduction

Hydrogen peroxide (H_2_O_2_) is a key intermediate in many signaling pathways in mammalian cells [1]. Its best-studied signaling effects are mediated by the oxidation of thiolates in transcription factors [2], kinases [3], [4] and phosphatases [5], [6], [7], [8]. However, whereas the redox active thiolates in these targets react with H_2_O_2_ with rate constants in the range of 9−164 M^−1^ s^−1^ [5], [6], [9], [1], the cell cytoplasm contains very abundant peroxiredoxins I and II (PrxI, PrxII) [10], [11], which react with H_2_O_2_ with rate constants in the range of 10^7^–10^8^ M^−1^ s^−1^ [12], [13]. And even in the absence of these and other catalysts, such as glutathione peroxidases and catalases, the ≈50μM glutathione thiolate in cells would outcompete those redox targets for H_2_O_2_ [14]. Explaining how those poorly reactive targets can be acted upon by H_2_O_2_ despite the strong competition by other agents is one enduring open question in redox biology [15], [16].

Three main types of explanations were proposed, which are not mutually exclusive [17]. First, there may be strong intracellular H_2_O_2_ concentration gradients. Sufficiently high H_2_O_2_ concentrations to oxidize the less reactive targets might be attained near very localized sites of H_2_O_2_ production or entry into the cytoplasm despite the mean cytoplasmic concentration being very low [15]. Indeed, estimates of H_2_O_2_ mean diffusion length [15] as well as recent mathematical models [18], [19] predict the occurrence of strong concentration gradients if H_2_O_2_ sources are localized. And observations with ratiometric H_2_O_2_ probes targeted to various cellular membranes support the existence of *microdomains* of elevated H_2_O_2_ concentration in the cytoplasm of live cells treated with growth factors [20]. However, despite the evidence for strong cytoplasmic H_2_O_2_ concentration gradients, the following question is still unclear. **Q1:** Are H_2_O_2_ concentrations attained near the supply sites sufficient to oxidize the above mentioned targets?

Second, peroxiredoxins may be transiently inactivated during signaling, allowing H_2_O_2_ to accumulate. The first such proposal was the floodgate hypothesis [21], which posits that the oxidation of the less reactive H_2_O_2_ targets is facilitated by the oxidation of eukaryotic 2-Cys peroxiredoxins to redox-inactive sulfinic and sulfonic forms (hyperoxidation). This hypothesis is supported by observations that overexpressing 2-Cys peroxiredoxins in mammalian cells blocks peroxide activation of nuclear factor kappa-light-chain-enhancer of activated B cells (NF-*κ*B) [22], [23], and that treatment of cells with tumor necrosis factor causes substantial hyperoxidation of PrxII [24]. Furthermore, it could explain why eukaryotic 2-Cys peroxiredoxins are much more susceptible to hyperoxidation than their bacterial homologues [21]. This susceptibility is due to structural features that are absent in the latter peroxiredoxins but widely conserved among the former ones [21], suggesting that it is selectively favored in eukaryotes. However, hyperoxidation was undetectable during mitogenic signaling [25], [26], [27], and unnecessary for ASK1 activation in H_2_O_2_-induced apoptosis [28]. More recently, it was hypothesized that localized hyperoxidation of the 2-Cys peroxiredoxins sharpens H_2_O_2_ concentration gradients, contributing to increased H_2_O_2_ concentrations near sites of H_2_O_2_ supply [29]. The localization of hyperoxidation could explain the difficulty in detecting it in the experiments above, but this *localized floodgate* hypothesis remains untested. Further support for the role of localized inhibition of the peroxidase activity of 2-Cys peroxiredoxins in redox signaling was provided by Woo et al. [27]. These authors showed that upon stimulation of a wide variety of cell lines by growth factor or immune receptors the peroxidase activity of PrxI associated to cell membranes is inhibited by phosphorylation. However, it is unclear if this localized inhibition can substantially affect local H_2_O_2_ concentrations in the presence of abundant PrxII, which also associates to the platelet-derived growth factor (PDGF) receptor upon PDGF stimulation [25], and when active cytoplasmic 2-Cys peroxiredoxins can readily diffuse to the inhibition sites. Although PrxI phosphorylation promotes PDGF-dependent tyrosine phosphorylation of cellular proteins [27], this does not necessarily imply a direct inhibition of protein tyrosine phosphatases (PTP) by H_2_O_2_. For instance, PrxI inhibition should lead to local accumulation of sulfenic or disulfide PrxII, which in turn could oxidize a PTP, thereby inhibiting it. Overall, the following question needs to be addressed. **Q2:** Can a localized inactivation of the 2-Cys peroxiredoxins cause a sufficient elevation of local H_2_O_2_ concentrations to directly oxidize the less reactive targets in a signaling time frame?

Third, the action of H_2_O_2_ may be mediated by redox relays whereby peroxiredoxins and/or peroxidases act as initial H_2_O_2_ sensors and then oxidize the end targets [15], [30], [31]. Such a relay was first described for the peroxiredoxin Orp1 and the transcription factor Yap1 in the yeast *Saccaromyces cerevisiae* [32], and other similar relays in yeasts were described meanwhile [33], [34], [35]. More recently, both PrxI [28] and PrxII [2], [36] were shown to engage in redox relays in the cytoplasm of mammalian cells. This is an attractive hypothesis that could also explain the specificity of redox signaling. However, the following question needs to be clarified. **Q3:** Can in general the oxidized forms of cytoplasmic 2-Cys peroxiredoxins accumulate sufficiently to outcompete H_2_O_2_ in oxidizing the less reactive targets?

Conversely to a role in relaying oxidizing equivalents to redox targets, peroxiredoxins can protect moderately reactive thiolates near sites of H_2_O_2_ supply against oxidation. Kang et al. [37] have shown that PrxII is recruited to the vascular endothelial growth factor (VEGF) receptor 2 (VEGFR2) upon VEGF stimulation of endothelial cells, protecting this receptor against oxidation of its Cys1199 and Cys1206 residues. More recently the same group [38] showed that in proliferating cells PrxI associates with the centrosome protecting it from H_2_O_2_ during interphase. It is then inhibited by phosphorylation during early mitosis, thereby facilitating oxidative inactivation of centrosome-bound phosphatases. **Q4:** But, under what conditions can a local accumulation of the 2-Cys peroxiredoxins protect reactive thiols in other proteins from oxidation and what extent of protection is achievable?

Mathematical modeling has proved useful in assessing previous hypotheses and suggest new ones about the operation of thiol redox systems [39], [16], [40], [18], [19], [41]. Here we draw on the present knowledge of the reactivity and cellular concentrations of 2-Cys peroxiredoxins, sulfiredoxin (Srx) and thioredoxin 1 (Trx1) [5], [6], [12], [13], [42], [9], [1] and apply an integrative computational approach to address the four questions raised above.

Our analyses show the following. At low to moderate localized H_2_O_2_ supply rates commensurate to mitogenic signaling, a sharp H_2_O_2_ concentration gradient with a length scale of ~0.3μm is established. However, local H_2_O_2_ concentrations remain insufficient to oxidize PTPs in a mitogenic signaling time frame (Q1). Further, only drastic inhibitions of the peroxiredoxins' peroxidase activity can have a substantial effect on the local H_2_O_2_ concentration near supply sites (Q2), and this comes at the cost of dampening the cytoplasmic H_2_O_2_ concentration gradient. On the other hand, the concentrations of sulfenic and disulfide forms of Prx exceed those of H_2_O_2_ by several orders of magnitude, and also show strong gradients. Redox targets able to react with these forms at rate constants $∼200\;M^{-1}\;s^{-1}$ could be oxidized in minutes near the H_2_O_2_ supply sites (Q3). The recruitment of peroxiredoxins to specific sites such as caveolae has a modest effect (∼20% decrease) on local H_2_O_2_ concentrations (Q4). However, it can have a dramatic effect on the local concentrations of oxidized forms of the peroxiredoxins, further helping these species outcompete H_2_O_2_ for oxidation of redox targets. Altogether, these results suggest that sulfenate and/or disulfide forms of Prx mediate the oxidation of redox targets within ∼1μm of H_2_O_2_ supply sites under conditions consistent with mitogenic signaling.

At higher H_2_O_2_ supply rates, the system can show a *hyperoxidation catastrophe*, in keeping with a recent analysis [43]. At a critical H_2_O_2_ supply rate Prx abruptly becomes almost fully hyperoxidized and the H_2_O_2_ gradient collapses, allowing H_2_O_2_ to penetrate deeply into the cell and oxidize some of the most reactive targets. Recovery from such a state can start only below a lower critical H_2_O_2_ supply rate (hysteresis) and proceeds over a period of hours. The global floodgate hypothesis may thus find its place as part of a stress response in this context.

## 2 Models and methods

**Fig. 1:**
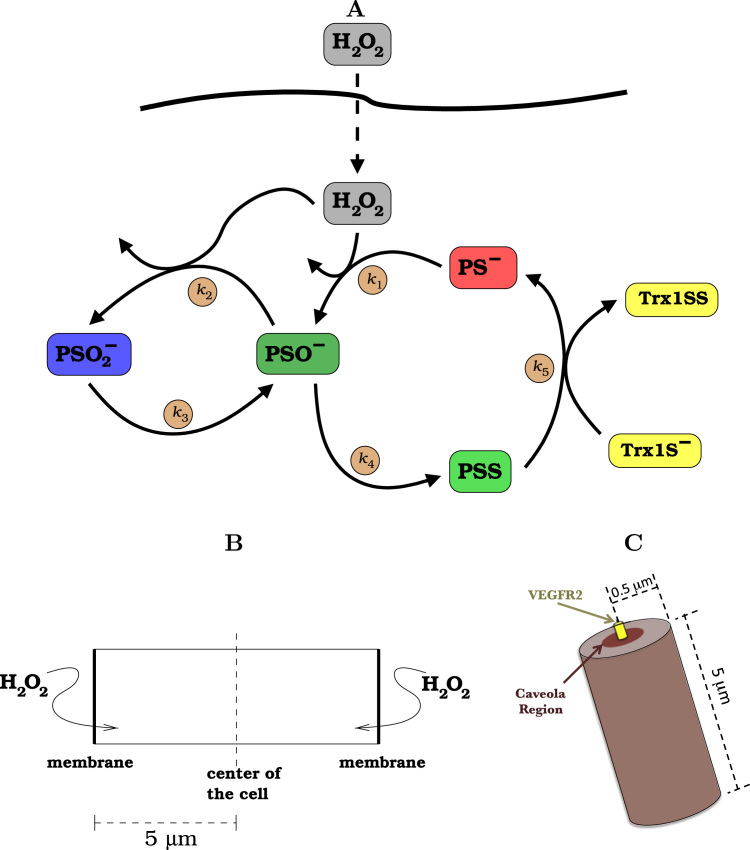
**Processes and geometry simulated: A:** H_2_O_2_ permeates the membrane with permeability constant $k_{p}$ and then oxidizes PS^−^ to PSO^−^ and PSO^−^ to PSO^−^_2_, with rate constants *k*_1_ and *k*_2_ respectively. PSO^−^_2_ is reduced back to PSO^−^ with rate constant *k*_3_ and PSO^−^ condenses with Prx's resolving Cys to yield PSS, with rate constant *k*_4_. Thioredoxin reduces PSS to PS^−^, with rate constant *k*_5_. **B:** We consider a section of a cell described by a rectangular prism with base area *A*, half-length 5 μm and a symmetry plane parallel to the top and bottom membranes. H_2_O_2_ is assumed to permeate both these membranes uniformly. This geometry models a cell in a cell layer and permits reducing the spatial problem to one dimension. Namely, along the normal to the plane of the permeant membranes. **C:** Scheme for the 3D simulation box of H_2_O_2_ concentration near a receptor. The region of the caveolae where the various peroxiredoxin forms can bind has a radius of 0.1 μm. The binding of 2% of the peroxiredoxin in the considered cylinder to this small region implies that the local concentration is 250-fold higher than in the bulk, considering a volume element with the depth of 10 nm. The H_2_O_2_ is able to enter through the top surface of the cylinder. In all other surfaces we implement a zero flux boundary condition for H_2_O_2_. With respect to the peroxiredoxin, all boundaries of this simulation box are set to zero-flux. The various forms of peroxiredoxins bound to the membrane are laterally immobile, and unbind with a spatially uniform rate constant. For parameter values please see Table 1.

The reference mathematical model implemented in this work focuses on the early stages of H_2_O_2_ signaling, and describes the processes and geometries depicted in Fig. 1. It embodies the following main simplifications. First, it neglects intracellular H_2_O_2_ sources. It is expectable that in early signaling H_2_O_2_ produced externally by NADPH oxidases is supplied to the cytoplasm mainly by permeation across the membrane [20]. Second, cytoplasmic H_2_O_2_ sinks other than 2-Cys peroxiredoxins are neglected. It is estimated [16], [18] that in most cells such sinks consume a small fraction of the H_2_O_2_, and their activity is insufficient to sustain substantial H_2_O_2_ gradients. Furthermore, recent quantitative proteomic studies of multiple human cell lines and tissues [11] consistently show that glutathione peroxidases are one to two orders of magnitude less abundant than PrxI and PrxII. The neglect of alternative H_2_O_2_ sinks is a conservative assumption with respect to the H_2_O_2_ attained in the cytoplasm. Third, it neglects thioredoxin oxidation, as the redox capacity of most cells is sufficient to keep thioredoxin reduced in absence of strong oxidative stress [16], [44], [40], [18]. Fourth, it accounts for a single 2-Cys peroxiredoxin whose concentration represents the sum of PrxI and PrxII, the two 2-Cys peroxiredoxins that are abundant in the cytoplasm of most mammalian cells [11]. The reference model considers that the single peroxiredoxin has the kinetic properties of PrxII. This is both because this peroxiredoxin has been more extensively characterized than PrxI, and because this is a conservative assumption with respect to most questions investigated in this work. The value we adopt for the rate constant for H_2_O_2_ reduction by thiolate peroxiredoxin ($k_{1}=1.0\times 10^{2}\;\mu M^{-1}\;s^{-1}$ [13]) is less conservative than that determined in ref. [45] ($13\;\mu M^{-1}\;s^{-1}$). However, even the former value has been determined at a sub-physiological temperature ($25^{o}C$), and is likely still conservative. In subsequent sections we will show that the main results are robust with respect to these assumptions.

The concentrations of the various chemical species in the cytoplasm are a function of both time and space, and therefore their evolution can be obtained through the following set of reaction diffusion equations:

(1) $$\begin{array}{cccc} \frac{\partial c_{H}}{\partial t} & = & D_{H}\nabla^{2}c_{H}-k_{1}c_{{\mathrm{PS}}^{-}}c_{H}-k_{2}c_{{\mathrm{PSO}}^{-}}c_{H},\\ \frac{\partial c_{PS^{-}}}{\partial t} & = & D\nabla^{2}c_{PS^{-}}-k_{1}c_{{{\mathrm{PS}}^{-}}^{}}c_{H}+k_{5}c_{PSS}c_{Trx},\\ \frac{\partial c_{PSO^{-}}}{\partial t} & = & D\nabla^{2}c_{PSO^{-}}+k_{1}c_{{\mathrm{PS}}^{-}}c_{H}-k_{2}c_{{\mathrm{PSO}}^{-}}c_{H} & \;+\;k_{3}c_{PSO_{2}^{-}}-k_{4}c_{PSO^{-}},\\ \frac{\partial c_{PSO_{2}^{-}}}{\partial t} & = & D\nabla^{2}c_{PSO_{2}^{-}}+k_{2}c_{{\mathrm{PSO}}^{-}}c_{H}-k_{3}c_{PSO_{2}^{-}},\\ \frac{\partial c_{PSS}}{\partial t} & = & D\nabla^{2}c_{PSS}+k_{4}c_{PSO^{-}}-k_{5}c_{PSS}c_{Trx}, \end{array}$$

where $c_{H}(r,t)$, $c_{{\mathrm{PS}}^{-}}(r,t)$, $c_{{\mathrm{PSO}}^{-}}(r,t)$, $c_{{\mathrm{PSO}}_{2}^{-}}(r,t)$, $c_{\mathrm{PSS}}(r,t)$, and $c_{\mathrm{Trx}}(r,t)$ are the concentrations of H_2_O_2_, of the peroxiredoxin forms, and of reduced thioredoxin, respectively. The reference values of the diffusion and rate constants are as in Table 1. The diffusion constant for all peroxiredoxin forms (MW=230kDa) was estimated from the experimentally determined diffusion constant for Immunoglobulin G (IgG, MW=153kDa) in the cytoplasm [46] by applying the expression

$$D_{\mathrm{Prx}}=D_{\mathrm{IgG}}\sqrt[{3}]{{\mathrm{MW}}_{\mathrm{IgG}}/{\mathrm{MW}}_{\mathrm{Prx}}},$$

as per the Stokes-Einstein relationship. The estimated value is of the same order of magnitude and just slightly higher than those recently determined for untranslating polysomes [47]. The diffusion constant for H_2_O_2_ [48] was determined for diffusion in buffer. The same authors determined a 5-fold lower diffusion constants for diffusion in hydrogels with a viscosity similar to that of the cell cytoplasm. Where pertinent we will discuss the consequences of the lower value of this diffusion constant. In Eqs. (1) we model the diffusion of the peroxiredoxin forms and of the H_2_O_2_ according to Fick's laws [49].

**Table 1:** **Model parameters**.

**Process**	**Rate**	**Parameter**	**Value**	**Ref.**
${\mathrm{PS}}^{-}+H_{2}O_{2}⟶{\mathrm{PSO}}^{-}+H_{2}O$	$k_{1}c_{H}c_{{\mathrm{PS}}^{-}}$	$k_{1}$	$1.0\times 10^{2}\;\mu M^{-1}\;s^{-1}$	[13]
${\mathrm{PSO}}^{-}+H_{2}O_{2}⟶{\mathrm{PSO}}_{2}^{-}+H_{2}O$	$k_{2}c_{H}c_{{\mathrm{PSO}}^{-}}$	$k_{2}$	$0.012\;\mu M^{-1}\;s^{-1}$	[42]
${\mathrm{PSO}}_{2}^{-}⟶{\mathrm{PSO}}^{-}$	$k_{3}c_{{\mathrm{PSO}}_{2}^{-}}$	$k_{3}$	$1.0\times 10^{-4}$ s^−1^	[40]
${\mathrm{PSO}}^{-}⟶\mathrm{PSS}$	$k_{4}c_{{\mathrm{PSO}}^{-}}$	$k_{4}$	1.7 s^−1^	[42]
$\mathrm{PSS}+\mathrm{Trx}1S^{-}⟶{\mathrm{PS}}^{-}+\mathrm{Trx}1\mathrm{SS}$	$k_{5}c_{\mathrm{PSS}}c_{\mathrm{Trx}}$	$k_{5}$	$0.21\;\mu M^{-1}\;s^{-1}$	[13]
Permeation of H_2_O_2_	$k_{p}A\;(c_{H}^{0}-c_{H}(0,t))$	$k_{p}$	$7.8\times 10^{-5}\;m\;s^{-1}$	See text
Diffusion constant of H_2_O_2_		$D_{H}$	$1.8\times 10^{-9}\;m^{2}\;s^{-1}$	[48]
Diffusion constant of peroxiredoxin		D	$5.2\times 10^{-13}$ m^2^ s^−1^	See text
Concentration of thioredoxin		$c_{\mathrm{Trx}}$	10μM	[11]

Per-cell H_2_O_2_ removal rate constants determined by Wagner et al. [50] allow to estimate the permeability constants in the range $10^{-7}-10^{-5}\;m\;s^{-1}$. In order to be very conservative about intracellular H_2_O_2_ concentrations, which in the models under appreciation are approximately proportional to the permeability constant, we adopt the higher value in Table 1.

The thioredoxin concentration is set at the constant value $c_{\mathrm{Trx}}(r,t)=10\;\mu M$, which is typical for mammalian cells [11]. We will also examine the effects of lower thioredoxin concentrations.

We solve these equations in the rectangular prism domain shown in Fig. 1B. The boundary conditions differ between the two ends of the domain: the membrane side at x=0μm, and the cell center side, at x=5μm. All concentration fields are solely function of the distance to the membrane. They have zero-flux boundary conditions except for the concentration of H_2_O_2_ at the membrane side of the domain. The amount of H_2_O_2_ that enters the domain through the membrane per unit time is $k_{p}A(c_{H}^{0}-c_{H}(0,t))$, where $c_{H}^{0}$ is the extracellular H_2_O_2_ concentration, and $c_{H}(0,t)$ is the intracellular H_2_O_2_ concentration adjacent to the membrane at time *t*. This condition is imposed as a boundary condition in the spatial derivative of $c_{H}$ at the membrane:

(2) $${\frac{\partial c_{H}}{\partial x}|}_{x=0}=-\frac{k_{p}}{D_{H}}(c_{H}^{0}-c_{H}(0,t)).$$

We use these boundary conditions to solve the Eqs. (1) in the considered domain, until we obtain the stationary profiles for the different concentration fields. The equations are integrated using a finite difference scheme with Δx=0.01μm, on the order of magnitude of the diameter of a peroxiredoxin dimer (∼8.0 nm, according to PdB file 4DSQ.pdb).

In order to analyze the potential protective effect of peroxiredoxin recruitment against oxidation of specific sites we consider two additional models. In the first one, we describe the binding of all peroxiredoxin species to the membrane by adding to Eqs. (1) the concentrations of the bound species. We consider that all peroxiredoxin forms have the same binding constant, $k^{+}$, to the membrane and the same unbinding constant, $k^{-}$. The ratio between the values of these constants is set such that 2% of the peroxiredoxin is bound to the membrane in the absence of H_2_O_2_ gradients. In this setting the stationary state does not depend on the numerical values of $k^{+}$ or $k^{-}$, only on their ratio. The mathematical model thus becomes:

(3) $$\begin{array}{lll} \frac{\partial c_{H}}{\partial t} & = & D_{H}\nabla^{2}c_{H}-k_{1}(c_{{\mathrm{PS}}^{-}}+\delta_{0}c_{{\mathrm{PS}}^{-}}^{s})c_{H}-k_{2}(c_{{\mathrm{PSO}}^{-}}+\delta_{0}c_{{\mathrm{PSO}}^{-}}^{s})c_{H}\\ \frac{\partial c_{{\mathrm{PS}}^{-}}}{\partial t} & = & D\nabla^{2}c_{{\mathrm{PS}}^{-}}-k_{1}c_{{\mathrm{PS}}^{-}}c_{H}+k_{5}c_{\mathrm{PSS}}c_{\mathrm{Trx}}+\delta_{0}(k^{-}c_{{\mathrm{PS}}^{-}}^{s}-k^{+}c_{{\mathrm{PS}}^{-}})\\ \frac{\partial c_{{\mathrm{PSO}}^{-}}}{\partial t} & = & \;D\nabla^{2}c_{{\mathrm{PSO}}^{-}}+k_{1}c_{{\mathrm{PS}}^{-}}c_{H}-k_{2}c_{{\mathrm{PSO}}^{-}}c_{H}+k_{3}c_{{\mathrm{PSO}}_{2}^{-}}-k_{4}c_{{\mathrm{PSO}}^{-}}\;+\delta_{0}(k^{-}c_{{\mathrm{PSO}}^{-}}^{s}-k^{+}c_{{\mathrm{PSO}}^{-}})\\ \frac{\partial c_{{\mathrm{PSO}}_{2}^{-}}}{\partial t} & = & D\nabla^{2}c_{{\mathrm{PSO}}_{2}^{-}}+k_{2}c_{{\mathrm{PSO}}^{-}}c_{H}-k_{3}c_{{\mathrm{PSO}}_{2}^{-}}+\delta_{0}(k^{-}c_{{\mathrm{PSO}}_{2}^{-}}^{s}-k^{+}c_{{\mathrm{PSO}}_{2}^{-}})\\ \frac{\partial c_{\mathrm{PSS}}}{\partial t} & = & D\nabla^{2}c_{\mathrm{PSS}}+k_{4}c_{{\mathrm{PSO}}^{-}}-k_{5}c_{\mathrm{PSS}}c_{\mathrm{Trx}}+\delta_{0}(k^{-}c_{\mathrm{PSS}}^{s}-k^{+}c_{\mathrm{PSS}}) \end{array}$$

$$\begin{array}{lll} \frac{{\mathrm{dc}}_{{\mathrm{PS}}^{-}}^{s}}{\mathrm{dt}} & = & -k_{1}c_{{\mathrm{PS}}^{-}}^{s}c_{H}(0,t)+k_{5}c_{\mathrm{PSS}}^{s}c_{\mathrm{Trx}}-k^{-}c_{{\mathrm{PS}}^{-}}^{s}+k^{+}c_{\mathrm{PS}{}^{-}}(0,t)\\ \frac{{\mathrm{dc}}_{{\mathrm{PSO}}^{-}}^{s}}{\mathrm{dt}} & = & k_{1}c_{{\mathrm{PS}}^{-}}^{s}c_{H}(0,t)-k_{2}c_{{\mathrm{PSO}}^{-}}^{s}c_{H}(0,t)+k_{3}c_{{\mathrm{PSO}}_{2}^{-}}^{s}-k_{4}c_{{\mathrm{PSO}}^{-}}^{s}-k^{-}c_{{\mathrm{PSO}}^{-}}^{s}+k^{+}c_{{\mathrm{PSO}}^{-}}(0,t)\\ \frac{{\mathrm{dc}}_{{\mathrm{PSO}}_{2}^{-}}^{s}}{\mathrm{dt}} & = & k_{2}c_{{\mathrm{PSO}}^{-}}^{s}c_{H}(0,t)-k_{3}c_{{\mathrm{PSO}}_{2}^{-}}^{s}-k^{-}c_{{\mathrm{PSO}}_{2}^{-}}^{s}+k^{+}c_{{\mathrm{PSO}}_{2}^{-}}(0,t)\\ \frac{{\mathrm{dc}}_{\mathrm{PSS}}^{s}}{\mathrm{dt}} & = & k_{4}c_{{\mathrm{PSO}}^{-}}^{s}-k_{5}c_{\mathrm{PSS}}^{s}c_{\mathrm{Trx}}-k^{-}c_{\mathrm{PSS}}^{s}+k^{+}c_{\mathrm{PSS}}(0,t) \end{array}$$

where $c_{{\mathrm{PS}}^{-}}^{s}(t)$, $c_{{\mathrm{PSO}}^{-}}^{s}(t)$, $c_{{\mathrm{PSO}}_{2}^{-}}^{s}(t)$ and $c_{\mathrm{PSS}}^{s}(t)$ are the volume concentrations of the species bound to the membrane. We assume that all the bound peroxiredoxin species have the same reactivity towards H_2_O_2_ and thioredoxin as the unbound counterparts. The binding of the peroxiredoxin species depends only on the concentration of the unbound species adjacent to the membrane ($c_{{\mathrm{PS}}^{-}}(0,t)$, $c_{{\mathrm{PSO}}^{-}}(0,t)$, $c_{{\mathrm{PSO}}_{2}^{-}}(0,t)$ and $c_{\mathrm{PSS}}(0,t)$). Accordingly, in Eqs. (3) the coefficient *δ*_0_ is equal to 1 next to the membrane and equal to 0 everywhere else.

In the second model for peroxiredoxin localization the same reaction-diffusion equations are solved within a cylindrical domain with azimuthal symmetry (Fig. 1C). In this setting, we consider that the binding of the peroxiredoxin species to the membrane can only occur in a circular region representative of a caveola. In this model, the bound species are not able to diffuse within the cell membrane. This system was simulated in the (*r*,*z*) plane with a second order finite difference scheme. The increments Δx used were identical to those in the 1D counterparts.

## 3 Results

### 3.1 Peroxiredoxins prevent deep penetration of H_2_O_2_ into the cell cytoplasm

**Fig. 2:**
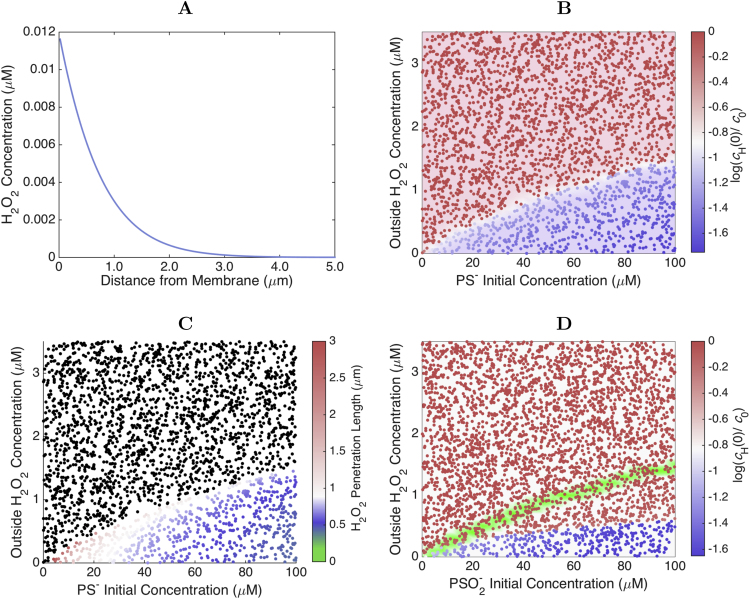
**H**_**2**_**O**_**2**_**penetration in the cell:** The H_2_O_2_ can penetrate the cell depending on the amount of cytoplasmic PS^−^ and on the extracellular H_2_O_2_ concentration. **A:** Concentration of H_2_O_2_ as a function of the distance from the membrane in the case of *c*^0^_PS_−=50 μM and *c*^0^_H_=0.4 μM. In this situation the total amount of H_2_O_2_ inside the cell is small and it is mainly located in the neighborhood of the membrane. **B:** Order of magnitude of the ratio between the H_2_O_2_ concentration adjacent to the membrane and the extracellular H_2_O_2_ concentration as a function of the initial cytoplasmic PS^−^ concentration and of the extracellular H_2_O_2_ concentration. Two regimes can be clearly observed: for high extracellular H_2_O_2_ concentrations the H_2_O_2_ oxidizes all the PS^−^ and invades the cell, while for low extracellular H_2_O_2_ concentrations the cytoplasmic PS^−^ is able to reduce the incoming H_2_O_2_. **C:** Except for low cytoplasmic PS^−^ concentrations, in the regime of low extracellular H_2_O_2_ concentrations the depth at which the cytoplasmic H_2_O_2_ concentration decays to 37% of its value near the membrane is in the range 0.5–0.7 µm, almost independently of $c_{\text{H}}$ and $c_{{\text{PS}}^{-}}$. Black dots indicate the collapse of the cytoplasmic gradient. **D:** Order of magnitude of the ratio between the H_2_O_2_ concentration adjacent to the membrane and the extracellular H_2_O_2_ concentration when initially all the peroxiredoxin is hyperoxidized. Each dot represents a separate simulation for the pertinent values of *c*^0^_H_ and initial peroxiredoxin concentration. The green shaded area marks the transition region between both regimes in panel B, highlighting the occurrence of hysteresis. (For interpretation of the references to color in this figure legend, the reader is referred to the web version of this article.)

We start by modeling the penetration of H_2_O_2_ in the cell for a given fixed concentration of extracellular H_2_O_2_, $c_{H}^{0}$. We first consider that all the peroxiredoxin is initially in reduced form. Under these conditions, for 2-Cys peroxiredoxin concentrations typical of most cells [11], the H_2_O_2_ concentration at steady state drops steeply near the membrane (Fig. 2A). Furthermore, the steady-state concentration of H_2_O_2_ adjacent to the membrane can be almost two orders of magnitude lower than the extracellular H_2_O_2_ concentration (Fig. 2B).

**Fig. 3:**
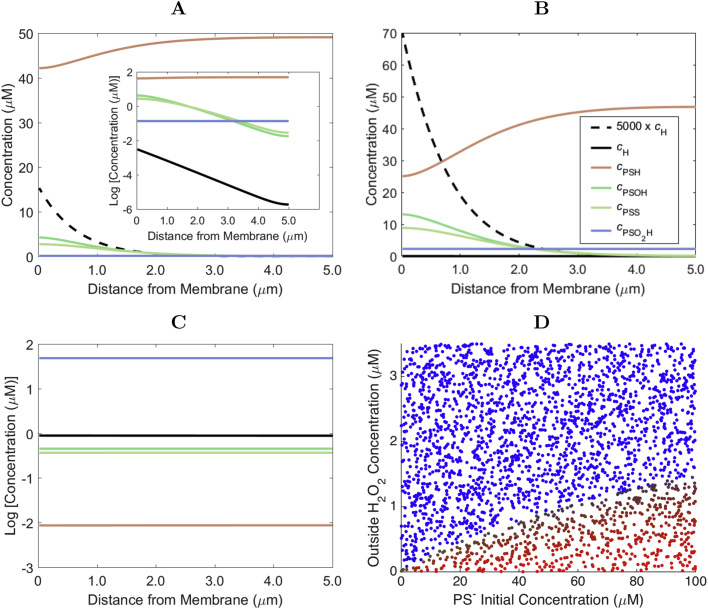
**Spatial distribution of peroxiredoxin species: A:** Concentration profiles of H_2_O_2_, PS^−^, PSO^−^, PSS and PSO^−^_2_ as a function of the distance from the membrane in the case of $c_{\text{PS}}-(0)\;=\;50\;\mu M$ and *c*^0^_H_=0.1 μM. For this low H_2_O_2_ influx rate, only a small fraction of PS^−^ is oxidized to PSO^−^ and PSS. The inset highlights the different orders of magnitude of the concentration of the various species and the strong gradients of $c_{{\text{PSO}}^{-}}$ and $c_{{\text{PSS}}^{}}$. The color code is as indicated in panel B. Note that in the main plot the concentration of H_2_O_2_ is multiplied by 5000 for visualization in the same scale. **B:** Same as A but for *c*^0^_H_=0.4 μM. In this case *c*_PS^--^_ is slightly depressed near the membrane, and $c_{{\text{PSO}}^{-}}$ and $c_{{\text{PSS}}^{}}$ are elevated. Due to the very low reduction rate constant for PSO^−^_2_, the concentration of this form becomes almost constant over the cytoplasm. **C:** Same as A but for *c*^0^_H_=1.0 μM. For these high extracellular concentrations, the H_2_O_2_ invades the cell and oxidizes most of the PS^−^ to PSO^−^_2_. **D:** Ratio between the average concentration of the various forms of peroxiredoxin and the initial concentration of PS^−^. Each point is colored according to the relative fractions of peroxiredoxin in each form. Pure red and blue indicate that 100% of the peroxiredoxin is in the PS^−^ or PSO^−^_2_ forms, respectively. Pure green indicates that 100% of the peroxiredoxin is in the PSO^−^+PSS forms. Comparing this figure to Fig. 2B, we observe that when the outside H_2_O_2_ concentration is very high, all the peroxiredoxin is hyperoxidized and the H_2_O_2_ is able to penetrate the whole cell. For low extracellular H_2_O_2_ concentrations, most of the peroxiredoxin is in its reduced form. (For interpretation of the references to color in this figure legend, the reader is referred to the web version of this article.)

This decay of the H_2_O_2_ concentration near the membrane is approximately exponential, similar to previous results for a simpler model [18], [19]. The typical width of the membrane neighborhood where H_2_O_2_ is concentrated has a weak dependence on both $c_{H}^{0}$ and the concentration of peroxiredoxin in thiolate form (Fig. 2C), as the following approximation also shows. For low $c_{H}^{0}$, only a small fraction of the cell's ${\mathrm{PS}}^{-}$ is oxidized, and therefore the steady state concentration of ${\mathrm{PS}}^{-}$ in the cell is much higher than that of the other redox forms (Fig. 3A). Therefore, the first equation in (1) can be approximated as $\partial_{t}c_{H}=D_{H}\partial_{x}^{2}c_{H}-k_{1}c_{H}c_{{\mathrm{PS}}^{-}}$, and $c_{{\mathrm{PS}}^{-}}$ is approximately equal to its initial value. The steady state for $c_{H}$ is then approximately given by

(4) $$c_{H}(x)∼c_{H}(0)\exp\left(-x/\lambda \right),$$

where

(5) $$c_{H}(0)∼\frac{c_{H}^{0}}{1+\frac{\sqrt{D_{H}k_{1}c_{{\mathrm{PS}}^{-}}}}{k_{p}}},$$

and

(6) $$\lambda =\sqrt{\frac{D_{H}}{k_{1}c_{{\mathrm{PS}}^{-}}}}$$

is the length scale of the decay of the H_2_O_2_ concentration. Considering a $c_{{\mathrm{PS}}^{-}}\approx 50\;\mu M$ and the parameter values in Table 1, one obtains λ≈0.60μm. Considering the 5-fold lower value of *D*_H_ obtained [48] for diffusion in a hydrogel with a viscosity approaching that of the cytoplasm one obtains λ∼0.27μm, which should be more representative of the situation *in vivo*. The order of magnitude of λ will be the same over the physiological range of peroxiredoxin concentrations, due to the shallow dependence of λ on $c_{{\mathrm{PS}}^{-}}^{-1/2}$.

### 3.2 Cytoplasmic H_2_O_2_ concentration gradients coexist with concentration gradients of sulfenic and disulfide peroxiredoxin forms

Importantly, coexisting with the cytoplasmic H_2_O_2_ gradients there are also strong concentration gradients of ${\mathrm{PSO}}^{-}$ and PSS (Fig. 3A, B). However, this is not the case of ${\mathrm{PSO}}_{2}^{-}$, whose concentration remains uniformly low throughout cytoplasm. The reasons for these differences among the spatial distributions of the various peroxiredoxin forms are as follows. H_2_O_2_ rapidly oxidizes ${\mathrm{PS}}^{-}$ to ${\mathrm{PSO}}^{-}$ near the membrane, and ${\mathrm{PSO}}^{-}$ is rapidly converted to PSS before is has time to diffuse far away. Reduction of PSS to ${\mathrm{PS}}^{-}$ is also too fast to permit this species to diffuse far away from the membrane. In turn, both the formation of ${\mathrm{PSO}}_{2}^{-}$ and its reduction to ${\mathrm{PSO}}^{-}$ are much slower, allowing ${\mathrm{PSO}}_{2}^{-}$ to diffuse throughout the cytoplasm.

In a narrow range of $c_{H}^{0}$ approaching the upper border of the blue region in Fig. 2B, the concentration of ${\mathrm{PS}}^{-}$ close to the membrane drops strongly (Fig. 3B) and the approximation of constant $c_{{\mathrm{PS}}^{-}}^{}$ breaks down. Due to the lower ${\mathrm{PS}}^{-}$ concentration, both the H_2_O_2_ concentration near the membrane (Fig. 2B) and the penetration length (Fig. 2C) increase in this range of $c_{H}^{0}$. However, the increase in the H_2_O_2_ concentration near the membrane in this range of $c_{H}^{0}$ is relatively modest.

### 3.3 Cytoplasmic H_2_O_2_ gradients collapse at high H_2_O_2_ supplies

As characterized more extensively elsewhere for a model neglecting concentration gradients [43], in cells where a high peroxiredoxin reduction capacity coexists with limited alternative H_2_O_2_ sinks the system shows bi-stability and hysteresis. This dynamic characteristic manifests itself as a run-away hyperoxidation of nearly all the peroxiredoxin and a drastic increase in cytoplasmic H_2_O_2_, once the extracellular H_2_O_2_ concentration exceeds a critical value ($c_{H}^{0*}$). Once this new steady state is established, recovery starts only after $c_{H}^{0}$ decreases below another lower critical value ($c_{H}^{0**}<c_{H}^{0*}$) and takes several hours. The reaction-diffusion model considered in the present work shows this behavior as well. When the extracellular H_2_O_2_ concentration reaches a critical value $\left(c_{H}^{0*}\right)$, the peroxiredoxin accumulates in hyperoxidized form (Fig. 3D). As a consequence, the cytoplasmic concentration gradients of H_2_O_2_, ${\mathrm{PS}}^{-}$, ${\mathrm{PSO}}^{-}$ and PSS collapse (Fig. 3C), and H_2_O_2_ concentrations become much higher throughout all the cytoplasm (Fig. 2B). The transition between these regimes is quite abrupt, and $c_{H}^{0*}$ increases approximately as the square root of the initial concentration of peroxiredoxin in the cell (Fig. 2B).

Remarkably, if one starts with a system where the peroxiredoxin is initially hyperoxidized, the critical values of $c_{H}^{0}$ $\left(c_{H}^{0**}\right)$ below which the peroxiredoxins are again able to sustain a H_2_O_2_ gradient are considerably lower than $c_{H}^{0*}$ (Fig. 2D). The system thus presents hysteresis.

Note that the actual values of $c_{H}^{0*}$ and $c_{H}^{0**}$ depend strongly on the permeability of the membrane and on the activity of alternative H_2_O_2_ sinks. Here we are neglecting these sinks and considering a high permeability constant (see Section 2). Therefore, we expect that in most cells these critical concentrations are higher than depicted in Fig. 2, Fig. 3.

### 3.4 The concentration of H_2_O_2_ near the membrane is insufficient to oxidize PTPs

Known rate constants for direct oxidation of PTPs by H_2_O_2_ are $k_{\mathrm{ox}}\leq 164\;M^{-1}\;s^{-1}$, which raises the question of whether H_2_O_2_ concentrations sufficient to directly oxidize PTPs are attainable in the cytoplasm. In order to frame this question, we first examine how the maximal cytoplasmic H_2_O_2_ concentrations relate to the extracellular concentrations, peroxiredoxin concentrations and cytoplasmic gradients to highlight a fundamental trade-off between signal localization and the maximal cytoplasmic H_2_O_2_ concentration. The latter concentration occurs adjacent to the membrane. Under conditions where only a small fraction of the peroxiredoxin is oxidized it is approximated by Eq. (5). The product $k_{\mathrm{sink}}=k_{1}c_{{\mathrm{PS}}^{-}}^{}$ in this equation represents the pseudo-first-order rate constant for H_2_O_2_ consumption in the cytoplasm, and therefore the following conclusions apply irrespective of the main cytoplasmic process contributing to $k_{\mathrm{sink}}$. Considering a generic eukaryotic cell with 10μm width, for signaling to be localized the length scale of the cytoplasmic H_2_O_2_ should be <1μm. As per Eq. (6) this implies $k_{\mathrm{sink}}>310\;s^{-1}$, conservatively considering $D_{H}=3.1\times 10^{-10}\;m^{2}\;s^{-1}$ as for H_2_O_2_ diffusion in a hydrogel [48]. Because even for the most permeable cells $k_{p}<10^{-5}\;m\;s^{-1}$, Eq. (5) implies $c_{H}(0)/c_{H}^{0}<1/30$. Therefore, in most cells the maximal cytoplasmic H_2_O_2_ concentrations are one to two orders of magnitude lower than the extracellular ones under conditions that warrant localized signaling. Furthermore, because $\sqrt{D_{H}k_{1}c_{{\mathrm{PS}}^{-}}^{}}/k_{p}⪢1$, for fixed $D_{H}$ and $k_{p}$ Eqs. (4), (5) imply that $c_{H}(0)\propto \lambda$. Hence, any decrease in $k_{\mathrm{sink}}$ will proportionately increase the length scale of the gradient. Moreover, the approximate proportionality of $c_{H}(0)$ to $k_{\mathrm{sink}}^{-1/2}$ implies that only very drastic inhibitions of cytoplasmic H_2_O_2_ clearance can have a substantial impact on the H_2_O_2_ concentrations near supply sites. For instance, in order increase $c_{H}(0)$ 2-fold Prx would have to be at least 75% inhibited.

**Fig. 4:**
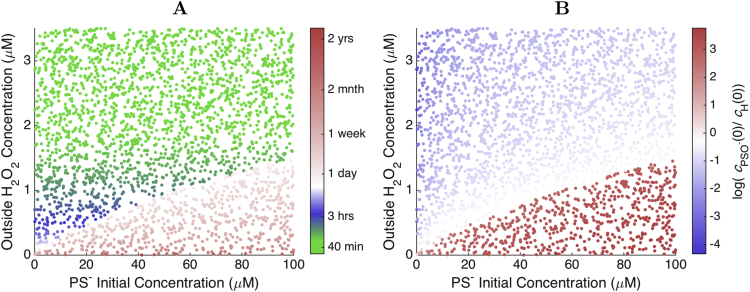
**H**_**2**_**O**_**2**_**capacity for signaling: A:** Minimum time scale for direct oxidation of PTPs by H_2_O_2_. The inverse of the pseudo-first-order rate constant for the direct oxidation of the active form of a PTP by H_2_O_2_ with *k_ox_*=164 M^-1^s^-1^ near the membrane is color coded as indicated. Values give the time needed to oxidize 63% of the target. Note the extremely slow oxidation under conditions that permit strong H_2_O_2_ gradients. Especially in the high hyperoxidation regime, times for oxidation are underestimated due to the neglect of alternative H_2_O_2_ sinks. **B:** Ratio between average intracellular concentrations of PSO^−^ and H_2_O_2_. Note the very high ratios (>1000) under conditions that permit strong H_2_O_2_ gradients. Results for the ratios between average intracellular concentrations of PSS and H_2_O_2_ are qualitatively similar. (The reader is referred to the web version of this article for a color version of this figure.).

Considering the $c_{H}(0)/c_{H}^{0}$ ratio estimated above, a $c_{H}^{0}=0.5\;\mu M$ would take ∼5.6 days to oxidize 63% of a very reactive PTPs residing at the cytoplasmic face of the membrane. Therefore, the results above indicate that in the regime where steep cytoplasmic gradients are maintained, H_2_O_2_ concentrations are far too low to directly oxidize PTPs in a mitogenic signaling time frame. Indeed, numerical results considering more generic conditions (Fig. 4A) fully substantiate this view: in this regime the oxidation of the PTPs could take weeks to years even at the points of highest H_2_O_2_ concentration. At higher $c_{H}^{0}$ values above $c_{H}^{0*}$ the H_2_O_2_ invades the cytoplasm, as per the previous section, and the time for oxidation decreases. [These results remain qualitatively the same if the higher condensation rate constant estimated for peroxiredoxin I [43] is considered (Fig. S1A).] However, although peroxisome-sequestered catalase and remaining glutathione thiolate cannot sustain a cytoplasmic H_2_O_2_ gradient where NADPH depletion prevents the action of peroxiredoxins and peroxidases, they can still substantially delay the oxidation of PTPs by H_2_O_2_ [14].

Although the results above pertain to a scenario where H_2_O_2_ permeates uniformly across all the membrane, the maximal H_2_O_2_ concentrations remain insufficient to quickly oxidize PTPs even in the extreme scenario where all the permeation is localized in a specific channel with a 50 nm diameter (Fig. S1B,C). Therefore, PTPs cannot be directly oxidized by H_2_O_2_ under conditions that avoid extensive peroxiredoxin hyperoxidation and keep the cytoplasm protected against excessive H_2_O_2_.

The hysteretic behavior described above is abolished if cells have (a) lower capacity to reduce PSS and/or (b) more-active alternative H_2_O_2_ sinks. In case (a) high extracytoplasmic H_2_O_2_ concentrations cause the cytoplasmic 2-Cys peroxiredoxins to accumulate in disulfide form instead (Fig. S2A), which is also accompanied by the collapse of the H_2_O_2_ gradients (Fig. S2B). In case (b) peroxiredoxin oxidation increases more gradually with the H_2_O_2_ supply rate, and the alternative H_2_O_2_ sinks may eventually be able to sustain a cytoplasmic H_2_O_2_ concentration gradient. However, cytoplasmic H_2_O_2_ concentrations will be even lower and oxidation of PTP by H_2_O_2_ even slower in this case.

### 3.5 Peroxiredoxins show good signaling properties for moderate H_2_O_2_ pulses

Interestingly, under all conditions where strong intracellular concentration gradients of H_2_O_2_ are sustained the concentrations of ${\mathrm{PSO}}^{-}$ and PSS exceed those of H_2_O_2_ by several orders of magnitude (Fig. 4B). As a consequence, these oxidized peroxiredoxin forms can oxidize PTPs and other redox targets much faster than H_2_O_2_ if their reactivity with these targets is comparable to that of H_2_O_2_.

**Fig. 5:**
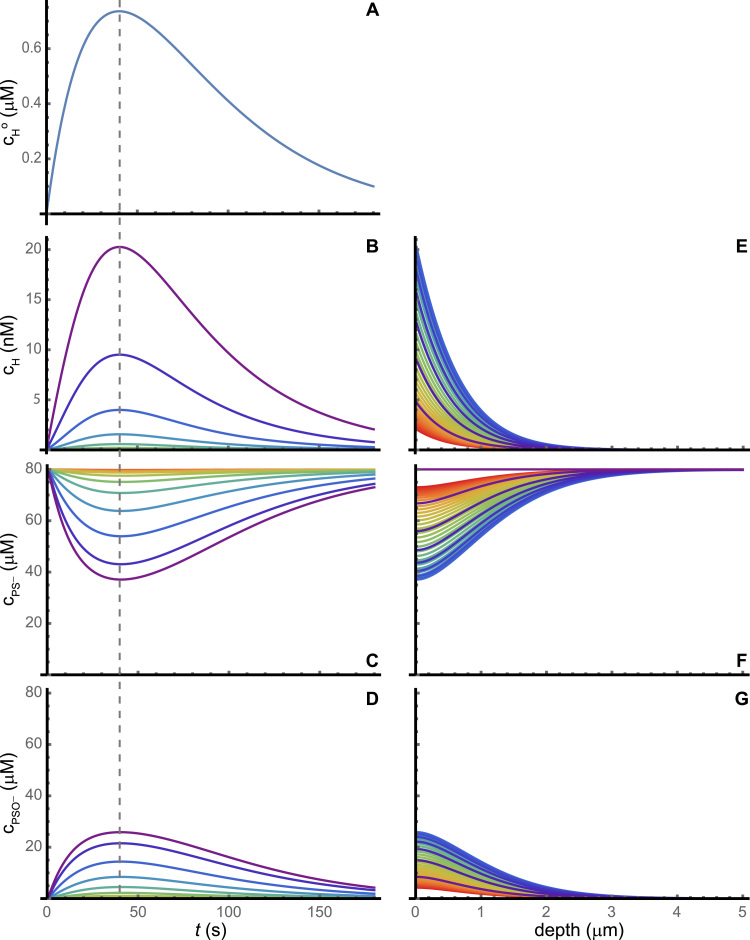
**Response to extracellular H**_**2**_**O**_**2**_**pulse. A:** Time course of the pulse. **B, C, D:** Time course of cytoplasmic concentrations of H_2_O_2_, PS^−^, and PSO^−^, respectively, at depths 0 (violet) to 5μm (red) from the membrane (0.5μm steps). **E, F, G:** Spatial profiles of cytoplasmic concentrations of H_2_O_2_, PS^−^, and PSO^−^, respectively, at times 0 (violet) to 180 s (red) from the membrane (5 s steps). The violet lines in panels E-G reflect the initial concentration change over depth. The time courses and spatial profiles for PSS are very similar to those for PSO^−^. Note the different scales for extracellular H_2_O_2_, cytoplasmic H_2_O_2_ and peroxiredoxin concentrations. The dashed gray line marks the time at which the cytoplasmic H_2_O_2_ concentration reaches its maximum. (For interpretation of the references to color in this figure legend, the reader is referred to the web version of this article.)

All the results presented so far pertain to situations where the extracellular H_2_O_2_ concentration is sustained long enough for the system to approach a steady state. However, H_2_O_2_ production is very dynamic in the seconds and minutes subsequent to growth factor binding to receptors [25], [27], [20]. Therefore, in order to further assess the signaling potentialities of ${\mathrm{PSO}}^{-}$ and PSS *vs.* H_2_O_2_ in this signaling context we simulated pulses of extracellular H_2_O_2_ concentration. The pulse shown in Fig. 5A attains a maximum extracellular H_2_O_2_ concentration of ≈0.75μM. The brief pulse causes little (<0.05%) hyperoxidation, but substantial peroxiredoxin oxidation (Fig. 5C,F). The results highlight two desirable properties of these peroxiredoxin forms as signaling intermediates. First, and as observed for the steady-state results, they attain much higher cytoplasmic concentrations than H_2_O_2_ (compare Fig. 5B,D). Second, they propagate the signal deeper into the cell than H_2_O_2_ (compare Fig. 5E,G). Namely, whereas the concentration of H_2_O_2_ decays near-exponentially with depth, high ${\mathrm{PSO}}^{-}$ and PSS concentrations are attained even 0.5μm from the membrane (Fig. 5D,G), allowing the oxidation of potential targets even at that depth.

These good signaling properties deteriorate for stronger pulses. A pulse attaining a maximum extracellular H_2_O_2_ concentration of 5.9μM already causes ≈35% hyperoxidation (Fig. S3E), and substantial H_2_O_2_ penetration in the cytoplasm (Fig. S3F). The concentrations of ${\mathrm{PSO}}^{-}$ and PSS start to decrease earlier than for the weaker pulse, and still in the ascending phase of the pulse, due to gradual hyperoxidation (Fig. S3D). The concentration gradients of these species are temporarily flattened out. Furthermore, the recovery of the peroxiredoxin redox status is strongly delayed, due to the slow reduction of ${\mathrm{PSO}}_{2}^{-}$ (Fig. S3C,D,E).

A pulse attaining a maximum extracellular H_2_O_2_ concentration of 12μM triggers the hyperoxidation “catastrophe” described above (Fig. S4). Virtually all peroxiredoxin is hyperoxidized within 1 min (Fig. S4E), all the cytoplasmic gradients collapse (Fig. S4F-H). Although maximal ${\mathrm{PSO}}^{-}$ and PSS concentrations similar to the previous pulse are attained at ≈10s after the onset of the pulse, the concentrations of these species fall precipitously thereafter and nearly vanish after ≈1min (Fig. S4D). Recovery of the peroxiredoxin redox state after the pulse occurs in a time scale of hours.

### 3.6 Peroxiredoxin recruitment causes modest local H_2_O_2_ depletion

**Fig. 6:**
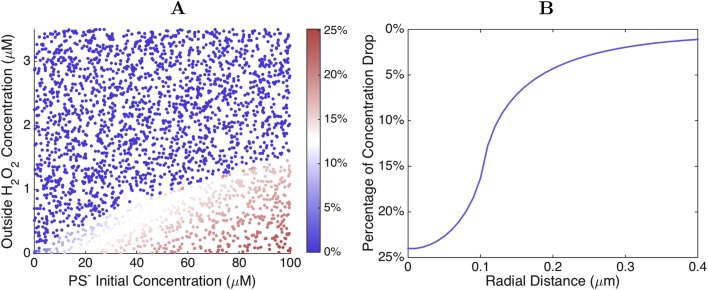
**Effects of peroxiredoxin recruitment: (1) Local H**_**2**_**O**_**2**_**depletion. A:** Decrease in H_2_O_2_ concentration near the membrane if 2% of the peroxiredoxin is uniformly distributed at the membrane. **B:** Decrease in H_2_O_2_ concentration near caveolae if 2% of the peroxiredoxin is concentrated at one caveola. For the explored range of conditions local H_2_O_2_ concentrations can decrease by up to 25%. (The reader is referred to the web version of this article for a color version of this figure.).

A small fraction (up to a few percent) of PrxI and PrxII is able to associate to the cellular membrane [51], [25], [27]. Moreover, PrxII is recruited to VEGFR2 upon VEGF stimulation of endothelial cells, protecting this receptor against oxidative inactivation by H_2_O_2_ [37]. The most straightforward explanation for this protection is that the co-localization of PrxII locally depletes H_2_O_2_. In order to assess the extent of local H_2_O_2_ depletion that is achievable in this way, we model two different scenarios. In the first scenario we consider that 2% of all the peroxiredoxin is reversibly and uniformly bound to the membrane, and that all redox states of peroxiredoxin show similar binding and unbinding rate constants. This system is described by Eqs. (3). Similarly to the system analyzed in the previous sections, the H_2_O_2_ concentration either drops exponentially near the membrane, or invades the cell, depending on the value of $c_{H}^{0}$ and on the total concentration of peroxiredoxin. The binding of this small fraction of peroxiredoxin to the membrane does not alter the critical values $c_{H}^{0*}$ (compare Figs. 6A and 3B). However, it decreases the concentration of H_2_O_2_ adjacent to the membrane under the conditions where the peroxiredoxin is able to sustain a strong cytoplasmic H_2_O_2_ concentration gradient (*i.e.*, where $c_{H}^{0}<c_{H}^{0*}$). This decrease is in the range of 10–25%, and is the stronger the higher the initial $c_{{\mathrm{PS}}^{-}}^{}$ and the lower $c_{H}^{0}$ are (Fig. 6A).

In the second scenario 2% of all peroxiredoxin is bound specifically to one caveola. We considered a cylindrical 3D simulation box as depicted in Fig. 1C. We ran the simulation in the regime of interest, *i.e.*, for high initial concentration of ${\mathrm{PS}}^{-}$ ($c_{{\mathrm{PS}}^{-}}(r,0)=80\;\mu M$) and relatively low $c_{H}^{0}$ (0.40μM). The 1D results for the previous scenario indicate that these parameter values yield a moderate protection of the receptor by the bound peroxiredoxins, with little ${\mathrm{PSO}}_{2}^{-}$ accumulation. In the current 3D scenario, the concentration of ${\mathrm{PSO}}_{2}^{-}$ is also negligible, and a steady state is achieved by 3 s of simulation time. In this simulation, the steady state depends strongly on the magnitude of the binding and unbinding constants. We chose values of $k^{+}=1.0\times 10^{4}\;s^{-1}$ and $k^{-}=39.2\;s^{-1}$, which guarantee the binding of 2% of the peroxiredoxin to the membrane.

**Fig. 7:**
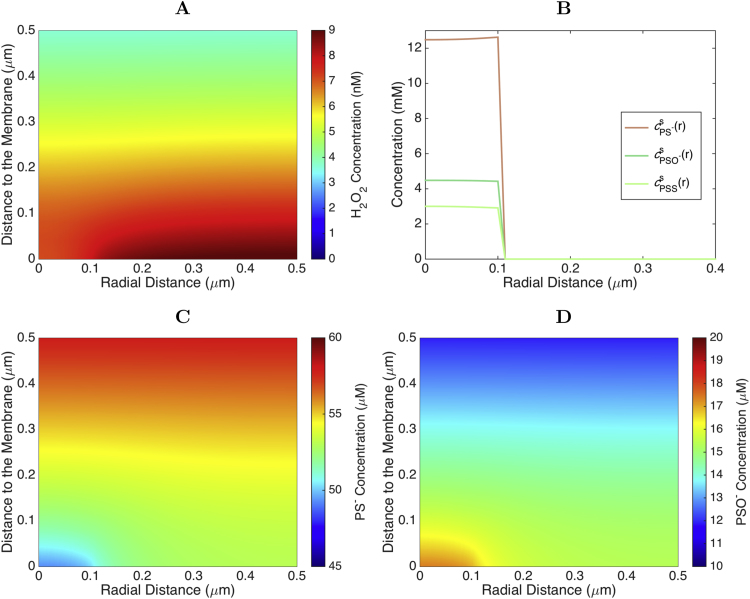
**Effects of peroxiredoxin recruitment (2): Distribution of H**_**2**_**O**_**2**_**and oxidized peroxiredoxin for the case where 2% of the peroxiredoxin is bound at one caveola. A:** Distribution of H_2_O_2_ close to the caveola. **B:** Distribution of the various peroxiredoxin forms at the membrane. **C:** Distribution of non-bound PS^−^ close to the caveola. We observe that in the caveola vicinity there is a minimum in the PS^−^ concentration. **D:** Distribution of non-bound PSO^−^ close to the caveola. The distribution of PSS is qualitatively similar. The concentrations of these peroxiredoxin forms is maximal next to the caveola. (The reader is referred to the web version of this article for a color version of this figure.).

Under these conditions H_2_O_2_ is appreciably depleted near the caveola (Figs. 6B and 7A). However, even this very strong concentration of peroxiredoxin at a caveola decreases the local H_2_O_2_ concentration adjacent to the membrane by no more than ≈25%, similar to the 1D simulation. This happens despite the local concentration of ${\mathrm{PS}}^{-}$ at the caveolae (≈10mM, Fig. 7B) strongly exceeding the concentration at the membrane in the previous scenario, which should apparently lead to a stronger protection of the receptor. However, as the ${\mathrm{PS}}^{-}$ at the caveloa is oxidized by incoming H_2_O_2_ the quick binding of new ${\mathrm{PS}}^{-}$ molecules depletes them in the cytoplasm around the caveola (Fig. 7C), owing to their relatively slow diffusion. In turn, this allows more H_2_O_2_ to diffuse from neighboring regions in the cytoplasm, thereby attenuating the effectiveness of protection. Remarkably, in the plane of the membrane not only the bound ${\mathrm{PS}}^{-}$ but also the bound PSS and ${\mathrm{PSO}}^{-}$ are strongly concentrated at the caveola region; that is, within 0.10μm from the receptor (Fig. 7B). Furthermore, the concentrations of unbound PSS and ${\mathrm{PSO}}^{-}$ are also elevated in the cytoplasm near the caveola (Fig. 7D). Therefore, the recruitment of peroxiredoxins to caveolae can strongly favor the peroxiredoxin-mediated oxidation of redox targets over their direct oxidation by H_2_O_2_.

## 4 Discussion

The results above address several key questions about H_2_O_2_ signaling and the participation of cytoplasmic 2-Cys peroxiredoxins in this process. Consistent with previous estimates [15], [40], models [18], [19] and experimental observations [20], we found that typical concentrations of 2-Cys peroxiredoxins are sufficient to sustain strong H_2_O_2_ concentration gradients in the cytoplasm of mammalian cells. As a result, in the absence of oxidative stresses able to extensively oxidize these peroxiredoxins most of the H_2_O_2_ in the cell cytoplasm is contained within a few tenths of μm of the supply sites (Fig. 2A,C). The existence of such steep H_2_O_2_ concentration gradients could explain how effective antioxidant protection can be achieved despite the high local H_2_O_2_ production rates involved in redox signaling [15]. However, the H_2_O_2_ concentrations attained even at the apex of these gradients are by and large insufficient to substantially oxidize PTPs in a mitogenic signaling time frame. Conservatively, we considered very high values for the H_2_O_2_ permeability constant and for the PTP oxidation rate constant, assumed diffusion constants for peroxiredoxin consistent with the mobility of its decameric form in a crowded cellular environment, and neglected alternative cytoplasmic H_2_O_2_ sinks. But even under these conservative assumptions oxidation of the PTPs would occur in a time scale of weeks (Fig. 4A) or longer. In an extreme scenario where all the H_2_O_2_ would permeate trough a single channel with 50 nm diameter the local H_2_O_2_ concentrations would oxidize PTPs in a time scale of several hours (Fig. S1B,C), still too slow for mitogenic signaling. This outcome ensues from the fundamental trade-off demonstrated in Section 3.4. Namely, given physiologically plausible H_2_O_2_ membrane permeabilities and diffusion constants, any agent consuming H_2_O_2_ fast enough to localize it within ≈1μm of a permeant membrane also generate a >30-fold trans-membrane gradient.

At moderately high H_2_O_2_ supply rates, peroxiredoxins can become substantially oxidized near the membrane, thus allowing for somewhat higher local H_2_O_2_ concentrations (Fig. 3B) at the cost of shallower cytoplasmic concentration gradients (Fig. 2C). However, the effect is modest (Fig. 2B,C) and restricted to a narrow range of H_2_O_2_ supply rates. These results also imply that the local inhibition of peroxiredoxins' peroxidatic activity – be it by hyperoxidation [21], phosphorylation [27] or non-covalent [40] – can only significantly increase local H_2_O_2_ concentrations if it substantially decreases the total cytoplasmic peroxidatic activity. Otherwise, the local inhibitory effect is efficiently counteracted by the diffusion of active peroxiredoxin from the bulk. However, whereas PrxI is inhibited by phosphorylation but not very susceptible to hyperoxidation, PrxII is susceptible to hyperoxidation but not inhibited by phosphorylation [27]. Because the concentrations of these two peroxiredoxins in the cytoplasm of mammalian cells are broadly similar [11], neither of these inhibitory mechanisms in isolation can dramatically increase local H_2_O_2_ concentrations. Indeed, it follows from Eq. (5) that a 50% decrease in total peroxidase activity will increase the H_2_O_2_ concentration near the membrane by at most $1-\sqrt{0.5}=29\%$. And as per Eq. (4) this will come at the cost of extending the length scale of H_2_O_2_ gradient by 29% as well.

At H_2_O_2_ supply rates slightly higher than those discussed in the previous paragraph, the peroxiredoxins become fully oxidized and H_2_O_2_ can then invade all the cytoplasm. But even in this situation where the interior of the cytoplasm would no longer be protected the oxidation of PTPs would be slow (Fig. 4A). A simple calculation shows that in order to oxidize 50% of the most reactive PTPs characterized to date ($k_{\mathrm{ox}}=164\;M^{-1}\;s^{-1}$ [6]) in 10 min a H_2_O_2_ concentration of $\log(2)/(164\;M^{-1}s^{-1}\times 600\;s)=7\;\mathrm{\mu M}$ would be required. The oxidation of other PTPs in the same time frame would require H_2_O_2_ concentrations in the order of 100μM. However, sustained 7μM *extracellular* H_2_O_2_ is sufficient to make Jurkat T cells apoptosise [52]. It is thus very unlikely that such high concentrations are attained anywhere in the cytoplasm.

**Fig. 8:**
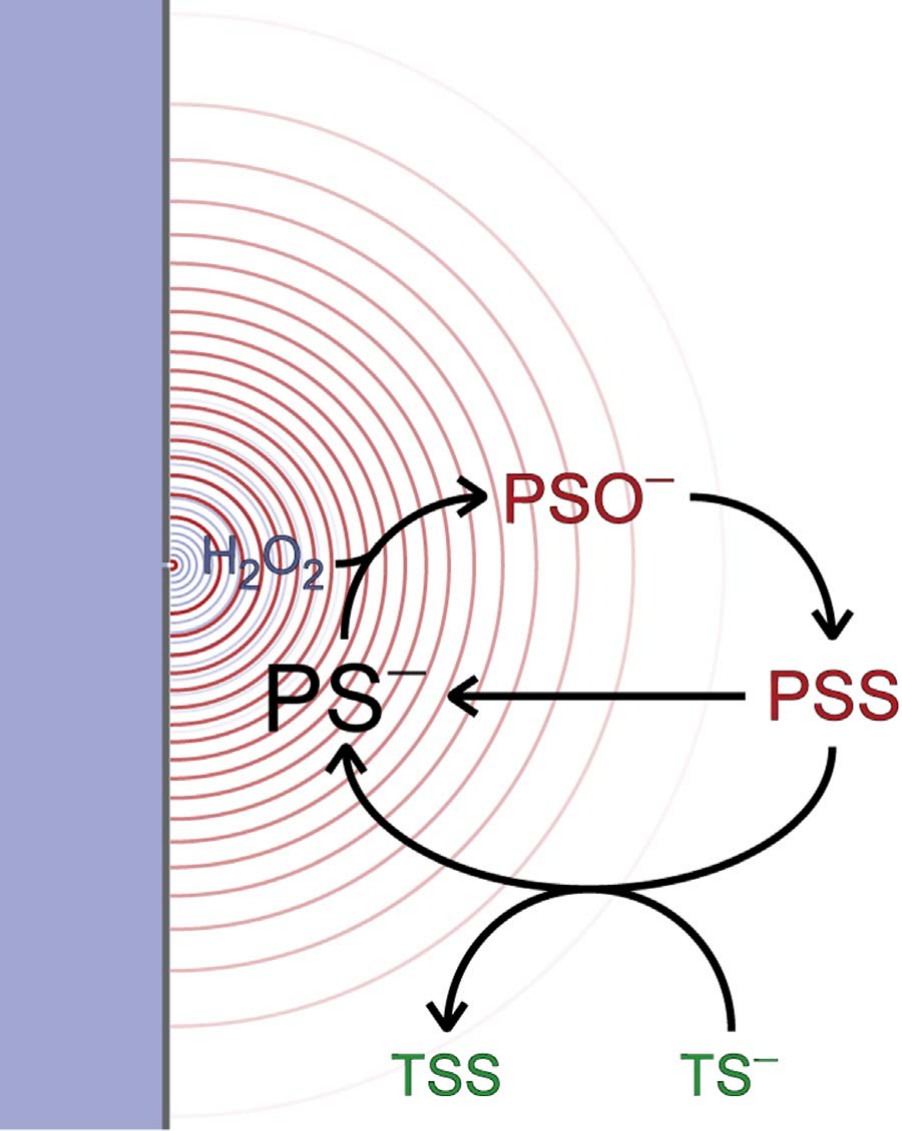
**H**_**2**_**O**_**2**_**signaling through localized redox relays**. At H_2_O_2_ (blue) supply rates commensurate with mitogenic signaling typical 2-Cys peroxiredoxins in the cytoplasm of mammalian cells sustain steep gradients but keep H_2_O_2_ concentrations very low throughout. This outcome is determined by the high cytoplasmic concentrations and high reactivity of these proteins. But these factors together with the limited rates at which the sulfenic and disulfide forms of these proteins (red) are reduced also determine that the concentrations of these forms strongly exceed those of H_2_O_2_. The oxidized peroxiredoxin species also show steep concentration gradients over the cytoplasm, here represented by level curves in red lines. Therefore, the oxidation of redox targets (green) mediated by them is also restricted to a vicinity of the H_2_O_2_ supply sites. But unlike the H_2_O_2_ concentration gradients (level curves in blue), those of the peroxiredoxin species plateau near the H_2_O_2_ supply sites, allowing the redox signal to penetrate slightly deeper into the cytoplasm. The competitive advantage of the peroxiredoxin sulfenic or disulfide forms over H_2_O_2_ in oxidizing targets at specific sites (*e.g.*, membranes, caveolae or endosomes) can be strongly amplified by localizing even a small fraction of the cytoplasmic peroxiredoxins to those sites. (For interpretation of the references to color in this figure legend, the reader is referred to the web version of this article.)

The sulfenic and/or disulfide forms of 2-Cys peroxiredoxins oxidize redox targets *in vitro* and *in vivo* forming redox relays [34], [35], [28], [2], [36]. Remarkably, the computational analyses presented here highlight three important advantages of peroxiredoxin-mediated oxidation of redox targets (*i.e.*, redox relays) over direct oxidation of the targets by H_2_O_2_. First, the concentrations of ${\mathrm{PSO}}^{-}$ and PSS exceed those of H_2_O_2_ by several orders of magnitude under conditions where H_2_O_2_ is contained near its supply sites (Fig. 4B). Therefore, even if these peroxiredoxin forms are just as reactive with the redox targets as H_2_O_2_ they can oxidize them much faster. However, the case of thioredoxin ($k_{5}=2.1\times 10^{5}\;M^{-1}\;s^{-1}$ [13]) illustrates that PSS can oxidize some targets at very high rate constants. Second, there are also important concentration gradients of these peroxiredoxin forms, thus retaining the capacity for localized signaling. Third, unlike the H_2_O_2_ gradients, those of ${\mathrm{PSO}}^{-}$ and PSS plateau at the membrane (Figs. 3A,B and 5G). This further favors the oxidation of the targets and allows the redox signal to propagate deeper into the cytoplasm. The latter may be important in some signaling contexts where the targets are located in intracellular membranes [53], [54]. The penetration of these peroxiredoxin forms in the cytoplasm is less problematic from the point of view of antioxidant protection than that of H_2_O_2_ because their interactions are expected to be specific. These considerations suggest a “localized redox relay” model of redox signaling (Fig. 8). This model is consistent with the observations by Klomsiri et al. [55] of localized formation of reversibly oxidized protein forms near H_2_O_2_ sources in LPA-stimulated PC3 and SKOV3 cells.

The localized oxidation of the Prx could regulate targets in at least the following four alternative ways. First, ${\mathrm{PSO}}^{-}$ and PSS can directly oxidize some targets [33], [34], [28], [35], [2]. Second, because these Prx forms can be glutathionylated (PSSG) [56], [57], PSSG might in turn glutathionylate redox targets either directly or mediated by glutaredoxin, as suggested in ref. [14]. S-glutathionylation is a well-established regulatory post-translational modification for many proteins, and this mode of operation would mirror similar proposals with glutathione peroxidases as peroxide sensors and glutaredoxins as key players [58], [59], [60], [61]. However, the presently available information about the kinetics of PSSG deglutathionylation does not permit to determine if this species is also localized. Third, the transfer of oxidizing equivalents to targets might also be mediated by Trx [62]. Fourth, a conformational change induced by Prx oxidation might either enable or hinder a non-covalent interaction with a target [63]. Kinetic experiments *in vitro* will be instrumental for testing if ${\mathrm{PSO}}^{-}$, PSS and/or PSSG can outcompete H_2_O_2_ for actuation of redox targets.

The results above highlight that despite small and very diffusible, H_2_O_2_ cannot diffuse far away from production sites due to the very high activity of cytoplasmic peroxiredoxins, and the redox signal is actually carried to distal sites by proteins.

Although under conditions of mitogenic signaling hyperoxidation has just a minor effect on local H_2_O_2_ concentrations and weakens the cytoplasmic concentration gradient, at higher H_2_O_2_ the opening of the hyperoxidation “floodgate” could be part of a stress response. The analysis in ref. [43] predicts that in cells with abundant peroxiredoxin reduction capacity and limited alternative H_2_O_2_ sinks the peroxiredoxin/thioredoxin/sulfiredoxin system shows bi-stability and hysteresis. The simulations in Fig. 2, Fig. 3 show that the predicted bi-stability and hysteresis persist when concentration gradients are taken into account. Furthermore, they show that the onset of the high-hyperoxidation state is accompanied by the collapse of the cytoplasmic H_2_O_2_ and peroxiredoxin concentration gradients, and by very low ${\mathrm{PSO}}^{-}$ and PSS concentrations despite the high H_2_O_2_ concentrations. Under these particular conditions, signal transmission through redox relays is interrupted and H_2_O_2_ may become able to oxidize some intermediate-reactivity targets deep into the cell. This predicted behavior is reminiscent of experimental observations for *Schizosaccharomyces pombe*, where exposure to high H_2_O_2_ concentrations caused strong hyperoxidation of the Tpx1 peroxiredoxin, interruption of redox-relay signaling to the Pap1 transcription factor and accumulation sufficient cytoplasmic H_2_O_2_ to trigger the stress response mediated by mitogen-activated protein kinase Sty1 [33].

A small fraction of the cytoplasmic 2-Cys peroxiredoxins can be recruited to the cellular membrane [51], [25], [37] or to the centrosome [38], where they can protect sensitive proteins against oxidation by H_2_O_2_ [37], [38]. Our simulations of the effects of localizing 2% of the cytoplasmic peroxiredoxin (Fig. 6, Fig. 7) show a modest (∼25%) decrease in local H_2_O_2_ concentrations despite the very high (up to 10 mM) local concentration of peroxiredoxin considered. Moreover, the effect is quantitatively similar irrespective of the peroxiredoxin binding uniformly to the membrane or just at caveolae. This is because the fast binding of ${\mathrm{PS}}^{-}$ to the membrane in replacement of oxidized molecules depletes ${\mathrm{PS}}^{-}$ from the neighboring cytoplasm, facilitating the inflow of H_2_O_2_ into this region.

On the other hand, the recruitment of peroxiredoxin to specific sites can originate very high local concentrations of ${\mathrm{PSO}}^{-}$ and PSS. This effect can be further amplified if the H_2_O_2_ supply is also localized at the same sites, for instance through permeation by aquaporins [64], [65]. Oxidation by ${\mathrm{PSO}}^{-}$ and PSS of targets also recruited to the same sites could thus be very fast. Likewise, a local inactivation of PrxI as described in ref. [27] although having a modest effect on local H_2_O_2_ concentrations could effectively block signal transmission through PrxI-mediated redox relays. Therefore, the possibility that the recruitment of peroxiredoxins to specific sites plays a more important role in facilitating and regulating the operation of localized redox relays than on modulating local H_2_O_2_ concentrations deserves further consideration. The observed protective effect of PrxII against VEGFR2 oxidation by H_2_O_2_ can as well be explained in this framework: the selective binding of oxidized PrxII, but not ${\mathrm{PrxIIS}}^{-}$, to VEGFR2 might protect the sensitive thiols in the latter protein by blocking access to H_2_O_2_ and/or by inducing a conformational change that hindered its reactivity.

Although the geometry addressed in this work applies most properly to situations where H_2_O_2_ permeates the apical and basolateral membranes of a cell in a cell layer, the qualitative results are valid for other geometries. Namely, for H_2_O_2_ release from endosomal vesicles or other intracellular membranes. Likewise, the key features determining the occurrence and functional advantages of localized redox relays over direct oxidation of targets by H_2_O_2_ are common to most eukaryotic cells known to date.

## Author contributions

AS, FSA, RDMT designed analyses; AB, FSA, PA, RDMT performed analyses; AS, RDMT wrote the paper.

## Competing financial interests

The authors declare no competing financial interests.

## Appendix A Supplementary data

Application 1

## Footnotes

Appendix A Supplementary data associated with this article can be found in the online version at 10.1016/j.redox.2017.01.003.
