# Supplementary material for: Localized redox relays as a privileged mode of cytoplasmic hydrogen peroxide signaling
Source: Redox Biol. 2017 Jan 6;12:233–45. doi: 10.1016/j.redox.2017.01.003 (PMC5339411; doi:10.1016/j.redox.2017.01.003)
Supplement: Application 1 [file mmc1.pdf]

Supplementary Information for

R.D.M. Travasso, F. Sampaio dos Aidos, A. Bayani, P. Abranches, A. Salvador.

"Localized Redox Relays as a Privileged Mode of Cytoplasmic Hydrogen Peroxide Signaling"

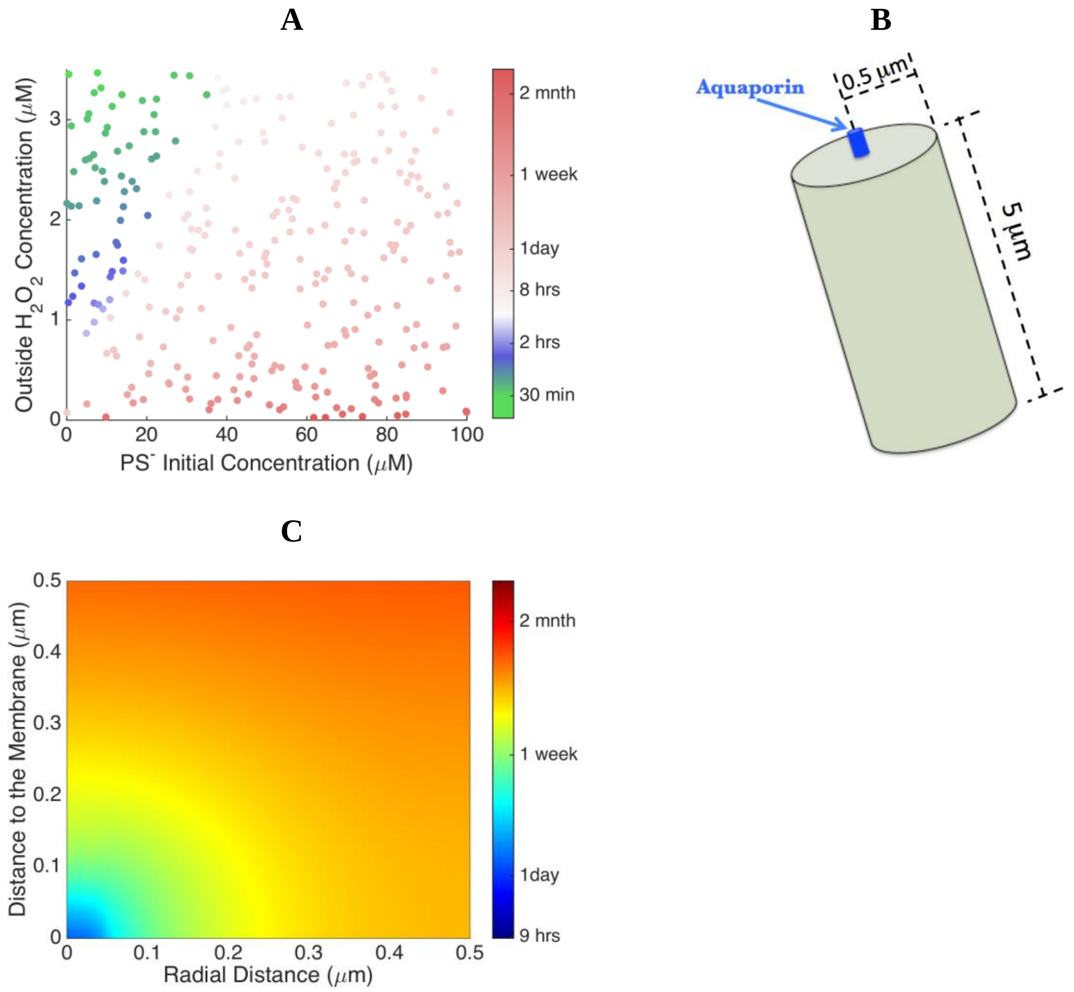

Figure S1. H_2O_2 capacity for signaling under various conditions. A: Minimum time scale for direct oxidation of PTPs by H_2O_2 for a condensation rate constant $k_4 = 88.7\ s^{-1}$, as estimated for peroxiredoxin I [1]. **B:** Scheme for the 3D simulation box near a membrane channel. **C:** Time scale for direct oxidation of a PTP reacting with H_2O_2 with a 164 $M^{-1}s^{-1}$ rate constant near a channel concentrating all the permeation rate of the membrane into a 50 nm diameter for an extracellular H_2O_2 concentration $c_H^0 = 0.40$ μM and $c_{PS^-}(0) = 80$ μM.

Reference

1. Selvaggio, G., Oliveira, V., Coelho, P. M. B. M., and Salvador, A. (2017) "Mapping the phenotypic repertoire of the cytoplasmic 2-Cys peroxiredoxin - thioredoxin system. 1. Design principles for effective analogic signaling." (submitted for publication).

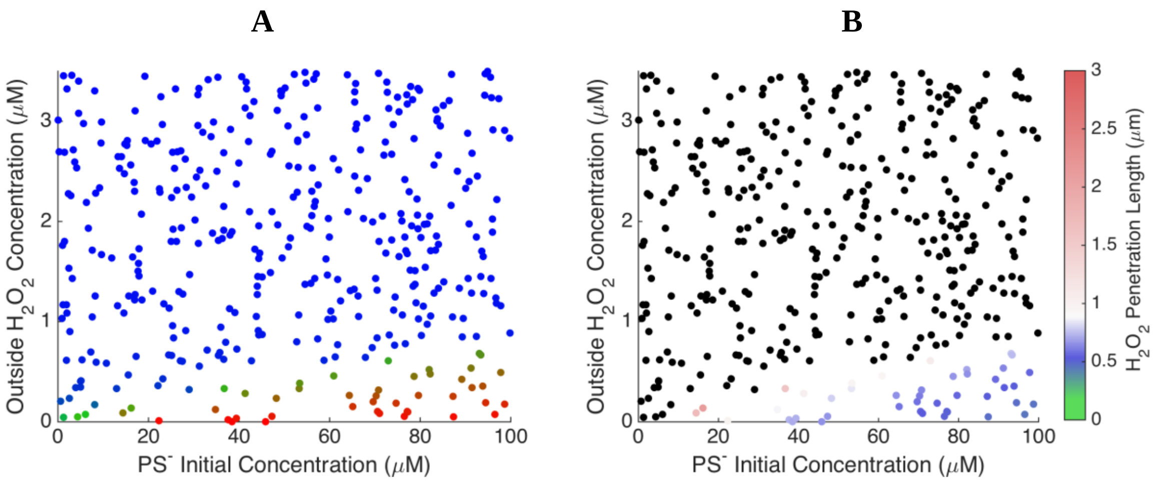

Figure S2. Dynamics of the system at low PSS reduction capacity. A: Ratio between the average concentration of the various forms of peroxiredoxin and the initial concentration of PS$^-$ for $c_{Trx} = 1\ \mu\text{M}$. Each point is colored according to the relative fractions of peroxiredoxin in each form following the same convention as in Figure 3D. Peroxiredoxins accumulate in disulfide form at high extracellular H_2O_2 concentrations. **B:** H_2O_2 penetration length for $c_{Trx} = 1\ \mu\text{M}$. Accumulation of the peroxiredoxins in disulfide form causes the cytoplasmic H_2O_2 concentration gradient to flatten out.

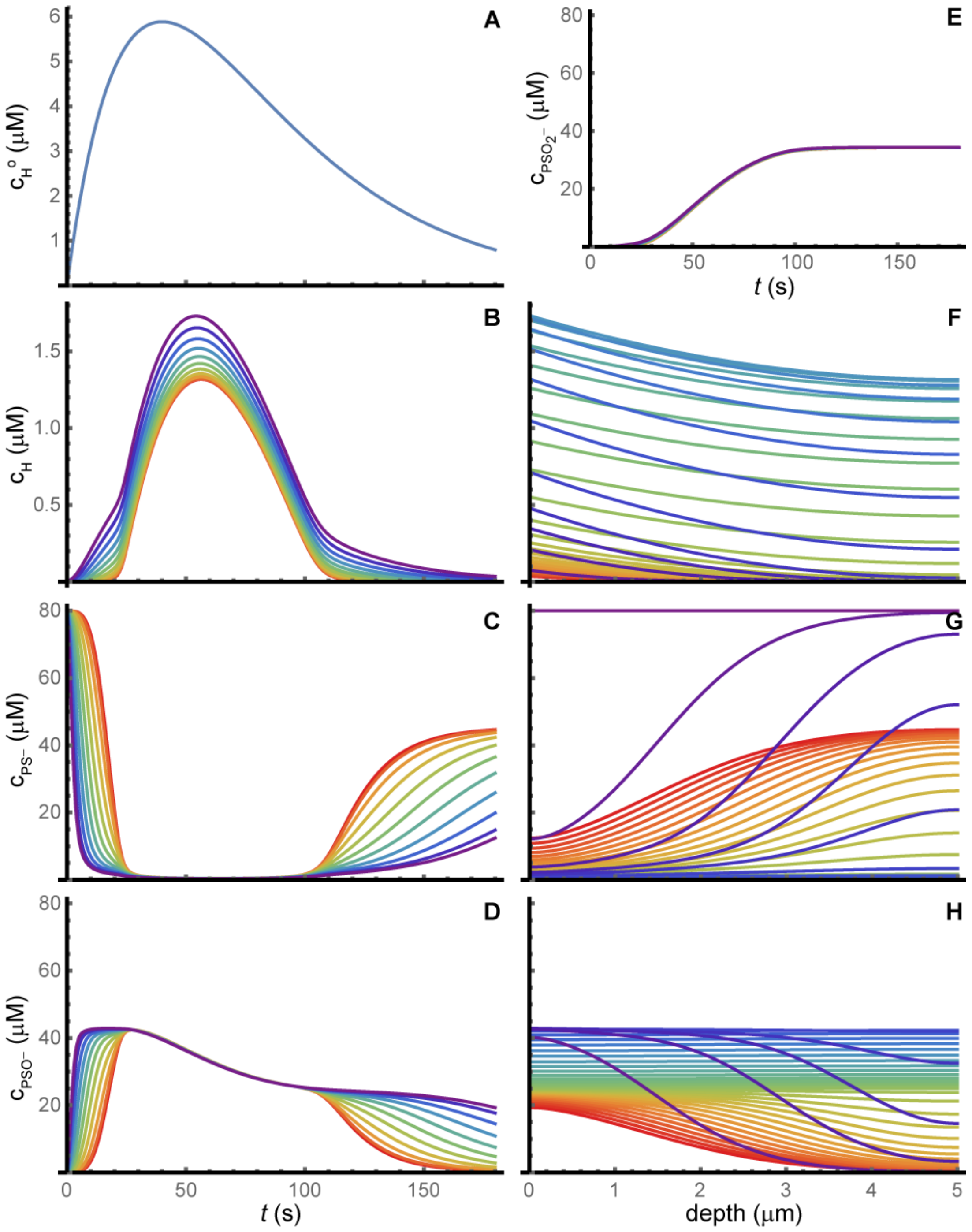

Figure S3. Response to extracellular 6 μM H_2O_2 pulses. A: Time course of the pulse. **B-E:** Time course of cytoplasmic concentrations of H_2O_2, PS^-, PSO^-, and $PSO_2{}^-$, respectively, at depths 0 (violet) to 5 μm (red) from the membrane (0.5 μm steps). **F-H:** Spatial profiles of cytoplasmic concentrations of H_2O_2, PS^-, and PSO^-, respectively, at times 0 (violet) to 180 s (red) from the membrane (5 s steps). The violet lines in panels E-G reflect the initial concentration change over depth. The time courses and spatial profiles for PSS are very similar to those for PSO^-. Note the different scales for extracellular H_2O_2, cytoplasmic H_2O_2 and peroxiredoxin concentrations.

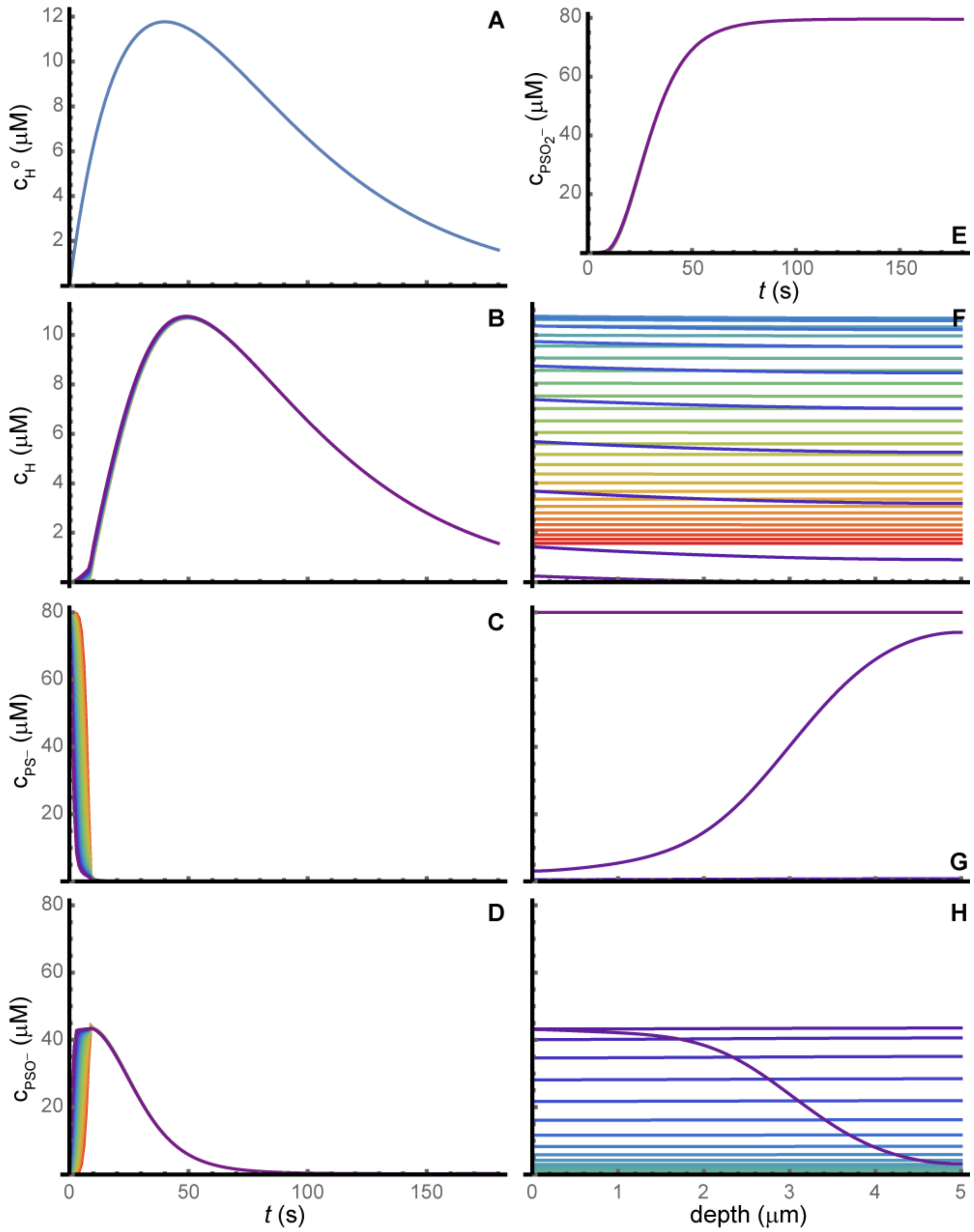

Figure S4. Response to extracellular 12 μM H_2O_2 pulses. A: Time course of the pulse. **B-E:** Time course of cytoplasmic concentrations of H_2O_2, PS^-, PSO^-, and PSO_2^-, respectively, at depths 0 (violet) to 5 μm (red) from the membrane (0.5 μm steps). **F-H:** Spatial profiles of cytoplasmic concentrations of H_2O_2, PS^-, and PSO^-, respectively, at times 0 (violet) to 180 s (red) from the membrane (5 s steps). The violet lines in panels E-G reflect the initial concentration change over depth. The time courses and spatial profiles for PSS are very similar to those for PSO^-. Note the different scales for extracellular H_2O_2, cytoplasmic H_2O_2 and peroxiredoxin concentrations.
